# The Neural Representation of a Repeated Standard Stimulus in Dyslexia

**DOI:** 10.3389/fnhum.2022.823627

**Published:** 2022-05-12

**Authors:** Sara D. Beach, Ola Ozernov-Palchik, Sidney C. May, Tracy M. Centanni, Tyler K. Perrachione, Dimitrios Pantazis, John D. E. Gabrieli

**Affiliations:** ^1^McGovern Institute for Brain Research, Massachusetts Institute of Technology, Cambridge, MA, United States; ^2^Program in Speech and Hearing Bioscience and Technology, Harvard University, Cambridge, MA, United States; ^3^Department of Speech, Language and Hearing Sciences, Boston University, Boston, MA, United States

**Keywords:** dyslexia, mismatch, repetition, adaptation, magnetoencephalography, multivariate pattern analysis, neural decoding

## Abstract

The neural representation of a repeated stimulus is the standard against which a deviant stimulus is measured in the brain, giving rise to the well-known mismatch response. It has been suggested that individuals with dyslexia have poor implicit memory for recently repeated stimuli, such as the train of standards in an oddball paradigm. Here, we examined how the neural representation of a standard emerges over repetitions, asking whether there is less sensitivity to repetition and/or less accrual of “standardness” over successive repetitions in dyslexia. We recorded magnetoencephalography (MEG) as adults with and without dyslexia were passively exposed to speech syllables in a roving-oddball design. We performed time-resolved multivariate decoding of the MEG sensor data to identify the neural signature of standard vs. deviant trials, independent of stimulus differences. This “multivariate mismatch” was equally robust and had a similar time course in the two groups. In both groups, standards generated by as few as two repetitions were distinct from deviants, indicating normal sensitivity to repetition in dyslexia. However, only in the control group did standards become increasingly different from deviants with repetition. These results suggest that many of the mechanisms that give rise to neural adaptation as well as mismatch responses are intact in dyslexia, with the possible exception of a putatively predictive mechanism that successively integrates recent sensory information into feedforward processing.

## Introduction

The neural response to a change in repetitive stimulation – the *mismatch response* – is a hallmark of central auditory processing, dependent not only on novel, “deviant” input, but on a sensory-memory trace of the preceding “standard” stimulus (Näätänen et al., 2005). Studies have suggested that individuals with dyslexia, a specific reading disability, have a deficit in implicit memory that limits their perceptual learning in repetitive contexts (Ahissar and Jaffe-Dax, 2018). In this study, we leveraged the theory that the mismatch response depends on forming a representation of an expected “standard” stimulus through repetition in order to ask whether the neural representation of a standard is impoverished in dyslexia.

The change-specific component of the cortical evoked response is termed the *mismatch negativity* (MMN) when measured with electroencephalography (EEG) and the *mismatch field* (MMF) or *magnetic MMN* (MMNm) when measured with magnetoencephalography (MEG) (for reviews, see Näätänen, 2001; Näätänen et al., 2007; Fitzgerald and Todd, 2020). Elicitation of the mismatch does not require the participant’s attention, only that the brain discriminate the deviant stimulus from the standard stimulus. The discovery that mismatch responses, usually calculated as (amplitude_deviant_ – amplitude_standard_), are diminished in some clinical populations, including individuals with dyslexia and young children at risk for dyslexia, has led to their widespread use in studies of auditory processing and cognition [reviewed in Näätänen et al. (2012)]. In these studies, the primary focus is on how the quality or quantity of stimulus deviance relates to the size of the mismatch (e.g., Kujala et al., 2006; Bonte et al., 2007; Noordenbos et al., 2012).

However, the flip side of change detection is regularity violation (Näätänen et al., 2012). It is possible that a relative insensitivity to repetition contributes to findings of an abnormal mismatch response in dyslexia, particularly for speech stimuli (Schulte-Körne and Bruder, 2010; Gu and Bi, 2020). Repetition can set the stage for an enhanced response to a subsequent novel stimulus *via* different mechanisms. For one, it can habituate the neural response in a primarily feedforward manner, such that the deviant brings fresh afferent activity and causes a release from adaptation (May and Tiitinen, 2010). For another, it can establish an internal model of expected stimulation, such that each expected repetition further reduces the neural response [*expectation suppression* (Todorovic et al., 2011)], while an unpredicted deviant triggers an increase in neural response known as *prediction error* (Wacongne et al., 2012).

Recent work from our lab suggests that neural and perceptual adaptation deficits in dyslexia (Ahissar et al., 2006; Perrachione et al., 2016; Peter et al., 2019) are attributable to abnormalities in the latter mechanism, and specifically to poor integration of predictions into intact feedforward processing (Beach et al., 2022). In that study, we measured neural responses to pairs of stimuli with orthogonal manipulations of the expectation of repetition (which was explicit) vs. the repetition itself. However, one question that this paradigm could not address is whether individuals with dyslexia are less able to extract “standardness” from the ongoing sensory environment, akin to a deficit in statistical learning (Gabay et al., 2015; Vandermosten et al., 2019). A second unanswered question is whether increasing standardness has a cumulative effect on the processing of subsequent stimuli. In a predictive coding framework (Rao and Ballard, 1999; Baldeweg, 2006), such a phenomenon might reflect successive updates to a model of stimulus consistency that underlies perceptual inference and learning.

**FIGURE 1:**
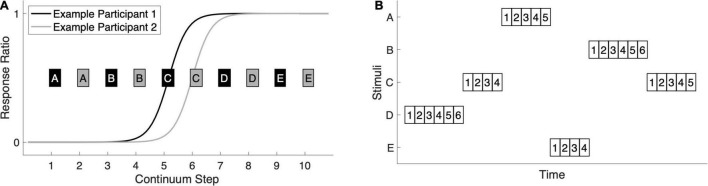
Stimuli and experimental design. **(A)** Stimuli were selected for each participant based on their behavioral responses in a categorical perception task in which participants heard, in pseudorandom order, 40 presentations of each of 10 stimuli forming a /ba/-/da/ continuum. They labeled each token as either ba or da with a button-press. We fit a logistic function to the response-ratio data to identify the location of the participant’s categorical boundary. The continuum step nearest the inflection point was selected as stimulus C and the other four stimuli were distributed evenly across the continuum. For Example Participant 1, whose sigmoidal fit is shown in black, the odd-numbered stimuli were selected (black boxes A–E). For Example Participant 2, whose sigmoidal fit is shown in gray, the even-numbered stimuli were selected (gray boxes A–E). **(B)** A schematic for the roving-oddball paradigm shows that trains of length four, five, or six were presented one after another for the duration of the experiment. Each train consisted of repetitions of the same stimulus (A–E), with the 1st presentation serving as the deviant and the 2nds through 6ths serving as the standards. The syllable stimuli were 310 ms in duration and the stimulus onset asynchrony was 575 ms. A total of 3,000 stimuli were presented in approximately 28 min, with all twenty possible standard-to-deviant stimulus transitions sampled.

To determine whether the neural processes that generate an increasingly robust standard representation are compromised in dyslexia, we analyzed MEG data recorded as adults with and without dyslexia (Table 1) were passively exposed to a roving-oddball paradigm composed of “trains” of consonant-vowel speech syllables (Figure 1). Each train consisted of four to six repetitions of the same stimulus. The first token in each train is thus always a deviant, and as it repeats, it becomes the new standard. Second through sixth presentations are considered standards at various levels of repetition. We used multivariate pattern analysis to decode the neural signature of standardness vs. deviance – what we will call the *multivariate mismatch* – with millisecond resolution. The outcome measures of interest were decoding *significance*, representing its reliability within and across individuals; decoding *accuracy*, representing the strength of standard-vs.-deviant information; and decoding *latency*, representing the efficiency with which the standard-vs.-deviant distinction arises.

**TABLE 1:** Behavioral characterization of the Control and Dyslexia groups.

		Control		Dyslexia		Difference		
Test	Subtest	Mean ± SD	Range	Mean ± SD	Range	*t*	*p*	*d*
KBIT-2	Matrices	114.75 ± 13.28	86–130	107.00 ± 15.25	86–132	1.88	0.07	0.54
WRMT-III	Word identification	110.17 ± 7.87	98–130	90.63 ± 9.39	77–113	7.82	<0.0001	2.26
	Word attack	101.50 ± 8.29	93–121	78.83 ± 9.95	59–107	8.58	<0.0001	2.48
	Listening comprehension	107.83 ± 6.75	99–120	102.17 ± 11.03	77–120	2.15	0.04	0.62
TOWRE-2	Sight word efficiency	108.63 ± 12.37	90–130	89.71 ± 9.42	73–111	5.96	<0.0001	1.72
	Phonemic decoding efficiency	104.29 ± 6.80	92–115	84.00 ± 8.50	61–97	9.13	<0.0001	2.64
GORT-5	Oral reading index	105.75 ± 8.76	84–121	85.08 ± 11.65	62–105	6.94	<0.0001	2.01
CTOPP-2	Elision	9.63 ± 1.91	4–12	7.92 ± 2.55	3–11	2.63	0.01	0.76
	Blending words	11.79 ± 2.30	8–16	10.75 ± 2.74	3–14	1.43	0.2	0.41
	Non-word repetition	8.79 ± 2.23	6–13	6.38 ± 1.58	3–10	4.33	<0.0001	1.25
WAIS-IV	Digit span total	10.79 ± 2.62	6–19	8.79 ± 2.40	5–16	2.76	0.008	0.80

*KBIT-2, Kaufman Brief Intelligence Test – Second Edition (Kaufman and Kaufman, 2004); WRMT-III, Woodcock Reading Mastery Tests – Third Edition (Woodcock, 2011); TOWRE-2, Test of Word Reading Efficiency – Second Edition (Torgesen et al., 2012); GORT-5, Gray Oral Reading Test – Fifth Edition (Wiederholt and Bryant, 2012); CTOPP-2, Comprehensive Test of Phonological Processing – Second Edition (Wagner et al., 2013); WAIS-IV, Wechsler Adult Intelligence Scale – Fourth Edition (Wechsler, 2008); d, Cohen’s d. All scores are age-based standard scores.*

We first lay out some of the characteristics of the multivariate mismatch: when it arises in the course of auditory processing, when it peaks, and how long it lasts. We use temporal generalization to describe its dynamics across the trial. We then explore the tradeoff between deviance and standardness in neural responses by determining how many repetitions are needed to make a standard that is distinguishable from a deviant. If dyslexia is characterized by a relative insensitivity to repetition, we might expect to see that a significant mismatch requires a more established standard in this group. Finally, we investigate the role of repetition history by comparing the strength of the mismatch across different levels of repetition. If the incorporation of predictions is reduced in dyslexia, we might expect to see that standardness accrues over repetitions in typical readers, but not in dyslexia.

## Results

### Standard-vs.-Deviant Decoding Is Robust in Control and Dyslexia

We first ensured that the roving-oddball paradigm (Figure 1) elicited neural responses to the deviant that were distinct from those evoked by all other (standard) stimuli combined. Given prior reports of diminished or absent mismatch responses in dyslexia, we compared the accuracy and latency of standard-vs.-deviant decoding between groups to determine if the multivariate analog of the mismatch was weaker or delayed in dyslexia. Decoding was performed on individuals’ MEG sensor-level data at 1-ms resolution using linear support vector machines, yielding a measure of standard-vs.-deviant classification accuracy over time.

**FIGURE 2:**
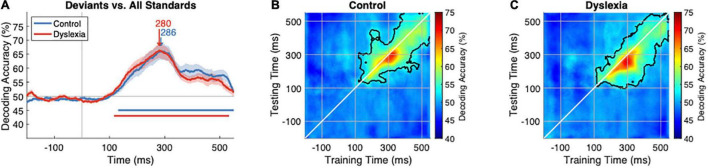
Standard-vs.-deviant decoding is similar in Control and Dyslexia. **(A)** Time course of decoding accuracy averaged over *n* = 24 Control (blue) and *n* = 24 Dyslexia (red) participants. For each participant separately and at each time point independently, a classifier was trained to distinguish deviant trials from standard trials, where deviants were the 1st tokens in a train and standards were the 2nd, 3rd, 4th, 5th, and 6th tokens. Data from all trials were included, so there were no stimulus differences between deviants and standards. Standard-vs.-deviant decoding is significantly above chance after 100 ms, as indicated by the horizontal lines in corresponding colors, and reaches its peak accuracy, on average, 282 ms after sound onset. **(B,C)** Temporal-generalization matrices averaged within the Control and Dyslexia groups. On the diagonal (white line) is the traditional decoding analysis, in which a classifier is trained and tested at each time point [corresponding to **(A)**]. Off the diagonal are decoding accuracies for classifiers trained at one time point (*x*-axis) and tested at every other time point (*y*-axis). Regions of significantly above-chance temporal generalization are outlined in black.

The average time course of decoding accuracy is plotted in Figure 2A, showing remarkable consistency across the groups from sound onset at 0 ms, where accuracy is near chance, until the traces begin to diverge slightly around 350 ms. A peak accuracy of 66% in each group is achieved at 280 ms in Dyslexia and 286 ms in Control. Two-sample cluster-corrected sign-permutation tests (cluster-defining threshold *p* < 0.05, corrected significance level *p* < 0.05) confirmed that the groups’ traces did not differ from one another. Moreover, neither the onset latency of significant decoding nor the latency of peak decoding accuracy differed between the groups (two-sample bootstrap tests). Thus, the multivariate mismatch to speech syllables appears similar in strength and timing in the two groups.

### Temporal Dynamics of the Multivariate Mismatch

Cluster-corrected sign-permutation tests (cluster-defining threshold *p* < 0.05, corrected significance level *p* < 0.05) were used to establish that decoding accuracy was above chance in each group, beginning around 120 ms and continuing for more than 400 ms (horizontal lines in Figure 2A; Control, *p* = 0.0002; Dyslexia, *p* = 0.0002). Because this effect was protracted in time, we sought to uncover some of the neural dynamics underlying the sustained decoding of deviants from standards by evaluating whether classifiers trained at one time point could generalize to other time points. The logic of the temporal-generalization approach is that, if the deviant evokes a series of distinct patterns of brain activity, a classifier optimized to discriminate stimuli at one time will be ineffective at other times in the trial. If activity is persistent, however, a classifier will successfully generalize to other times in the trial.

Temporal generalization matrices, with classifier training time on the *x*-axis and classifier testing time on the *y*-axis, are shown in Figure 2B (Control) and Figure 2C (Dyslexia). The white diagonal line indicates the traditional decoding analysis in which a separate classifier is trained and tested at each time point independently. Off-diagonal decoding indicates generalization across time, with significant periods outlined in black (one-sided sign-permutation tests, cluster-defining threshold *p* < 0.05, corrected significance level *p* < 0.05). Significant generalization began shortly after 100 ms in both groups and lasted throughout the trial (Control, *p* = 0.0002; Dyslexia, *p* = 0.0002). The broad diagonal pattern observed in both Control and Dyslexia denotes successive phases of activity with a degree of maintenance over time.

The average generalization matrix of each group also demonstrated an asymmetrical pattern of off-diagonal decoding (red regions in Figures 2B,C), such that classifiers trained around 300 ms robustly generalized backward in time for approximately 50–100 ms. Classifiers trained around 250 ms did generalize forward in time, but with lower accuracy. This asymmetry likely reflects an increase in neural activity causing an improvement in the signal-to-noise ratio (SNR) from 250 to 300 ms, as accuracy is higher when a classifier is trained on high-SNR data and tested on low-SNR data than the converse (King and Dehaene, 2014). Overall, these results indicate that the neural activity that is unique to the deviant is both prolonged in time and dynamically changing over time.

### Standard vs. Deviant Is Robustly Decoded in Control and Dyslexia at All Levels of Repetition

To determine how deviance declines and standardness emerges with repetition, we trained linear support vector machines to classify whether trials were standard or deviant. Across five separate decoding analyses, we varied the repetition history of the standards, i.e., whether they were 2nd, 3rd, 4th, 5th, or 6th repetitions of the same stimulus. The deviants were held constant across analyses: they were always the 1st stimulus in each train, representing a change from the previous train of stimuli. Classifiers were trained and tested at each time point independently and within each participant separately, and thus we obtained one time course of decoding accuracy per participant per analysis. To evaluate whether decoding accuracy was above the 50% chance level in each participant group, we performed one-sided sign-permutation tests on the subject-specific time courses, with familywise error controlled across time points with cluster-based inference. If, for example, 2nd repetitions retain elements of deviance in their neural representations, we would expect the classifier to be unable to distinguish them from 1st presentations. Additionally, if individuals with dyslexia are less sensitive to repetition, we would expect to see that significant decoding only obtains after more repetitions of the standard.

**FIGURE 3:**
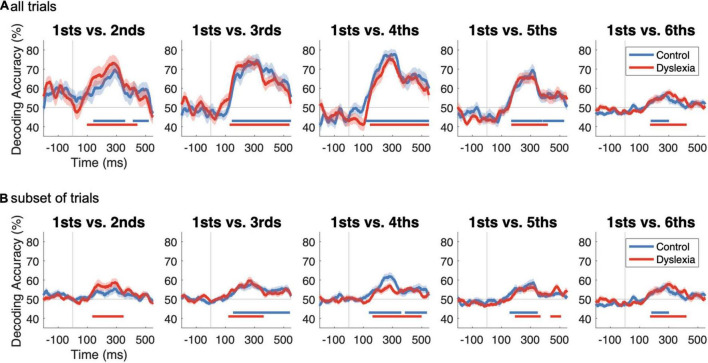
Decoding deviant vs. standard as a function of repetition history and number of trials. **(A)** Time courses of decoding accuracy as a function of the level of repetition of the standard. In each subplot, classifiers were trained to distinguish deviant trials from standard trials, where standards were, from left to right, the 2nd, 3rd, 4th, 5th, or 6th tokens in their train. Within-participant decoding results are shown as group averages (Control, blue; Dyslexia, red). Time points of significantly above-chance decoding are indicated by horizontal lines in corresponding colors below the traces (one-sample sign-permutation tests with cluster-based correction). No significant differences between groups in accuracy were identified (two-sample sign-permutation tests with cluster-based correction). **(B)** As in **(A)**, but in order to investigate the effect of trial numbers on decoding accuracy, we took a random subset of each participant’s 2nd, 3rd, 4th, and 5th standard trials equal to their number of usable “6th” before repeating the decoding analysis. This procedure significantly reduced decoding accuracy in every case [sign-permutation tests on differences from **(A)** to **(B)**].

Figure 3A shows that significant decoding was obtained in all five cases for both groups, indicating that the neural representation of “standardness” has already replaced “deviance” during the 2nd presentation of a stimulus. Significant time points are indicated by horizontal lines in corresponding colors below the traces (one-sample sign-permutation tests, cluster-defining threshold *p* < 0.05, corrected significance level *p* < 0.05; Control *p*’s between 0.0002 and 0.039; Dyslexia *p*’s between 0.0002 and 0.0004). The time courses show a steep rise in decoding accuracy beginning around 100 ms after stimulus onset, reaching peak accuracies of 70–80% around 300 ms. Accuracies then reach a lower plateau between approximately 400 and 500 ms before declining sharply. As suggested by the groups’ similar traces in each panel of Figure 3A, decoding accuracy was not significantly higher in one group than the other for any of the five analyses (two-sample sign-permutation tests, cluster-defining threshold *p* < 0.05, corrected significance level *p* < 0.05). This indicates that the multivariate mismatch is similarly robust in the two groups.

### The Strength of the Multivariate Mismatch Depends on the Number of Trials

The relatively lower decoding accuracies for 1sts vs. 5ths and 1sts vs. 6ths, in which standardness should be robustly established, was contrary to predictions. We suspected that these lower accuracies were because, compared to 2nds, 3rds, and 4ths, there were fewer 5ths and still fewer 6ths with which to train the classifier (Table 2), and this resulted in noisier and less accurate classification. We tested our hypothesis by repeating the decoding after randomly subsetting each participant’s 2nd, 3rd, 4th, and 5th trials to match their number of usable (after artifact rejection) 6th trials.

**TABLE 2:** Number of usable trials by type and group.

		Presented	Remaining after artifact rejection						
			Control		Dyslexia		Difference		
Type	Subtype		Mean ± SD	Range	Mean ± SD	Range	*t*	*p*	*d*
Deviant	1sts	600	576.75 ± 51.31	340–599	590.04 ± 12.00	554–600	1.24	0.22	0.36
Standard	2nds	600	577.25 ± 50.45	345–599	589.17 ± 12.97	546–600	1.12	0.27	0.32
Standard	3rds	600	577.08 ± 50.93	342–600	589.54 ± 12.28	557–600	1.17	0.25	0.34
Standard	4ths	600	576.75 ± 48.44	355–599	589.75 ± 13.20	548–600	1.27	0.21	0.37
Standard	5ths	400	385.83 ± 31.61	241–400	393.38 ± 7.71	367–400	1.14	0.26	0.33
Standard	6ths	200	193.25 ± 15.84	121–200	196.38 ± 4.08	183–200	0.94	0.35	0.27

*d, Cohen’s d.*

As anticipated, this procedure substantially reduced decoding accuracy, although it remained above chance in all but one case (1sts vs. 2nds in Control) (Figure 3B). Paired comparisons of the corresponding traces in Figures 3A,B revealed, in all cases, significantly lower decoding accuracy when the number of standard trials was reduced (one-sample sign-permutation tests, cluster-defining threshold *p* < 0.05, corrected significance level *p* < 0.05; 4 tests each in Control and Dyslexia; *p*’s between 0.028 and 0.0002). This analysis suggests that decoding metrics should not be compared across analyses when the contributing numbers of trials are not well matched. Therefore, subsequent analyses focused on 2nd, 3rd, and 4th standards, for which trial numbers were approximately equal (Table 2).

### Standard-vs.-Deviant Information Arises and Peaks at Similar Latencies in Both Groups

**FIGURE 4:**
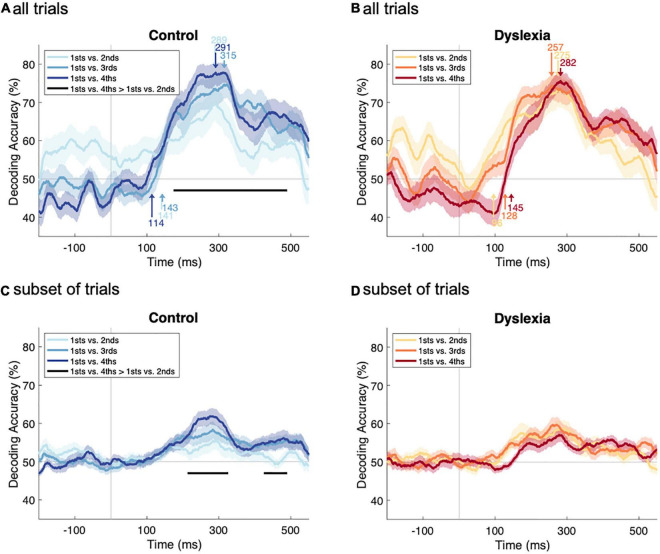
Comparing deviant-vs.-standard decoding as a function of repetition history. **(A,B)** Decoding-accuracy time series from Figure 3A (all trials), overlaid for easier comparison within Control **(A)** and Dyslexia **(B)** groups. Arrows in the lower portion of each panel indicate the onset latencies, in ms, of significant deviant-vs.-standard decoding. Arrows in the upper portion of each panel indicate latencies, in ms, of peak decoding accuracy. In Control, there is a significant difference in accuracy between 1sts-vs.-4ths decoding and 1sts-vs.-2nds decoding. **(C,D)** Decoding-accuracy time series from Figure 3B (subset of trials), overlaid for easier comparison within Control **(C)** and Dyslexia **(D)** groups. With reduced trial numbers, there is again, in Control, a significant difference in accuracy between 1sts-vs.-4ths decoding and 1sts-vs.-2nds decoding.

We next evaluated the possibility that standard-vs.-deviant information is slower to arise in Dyslexia, which is suggested by previous findings of significantly delayed onset and peak latencies of the MMN (Baldeweg et al., 1999; Mittag et al., 2013). We compared the onset latency of significant decoding as well as the latency of peak decoding between the groups at three levels of standard repetition. Onset latencies were derived from cluster-corrected sign-permutation tests as in Figure 3A and are labeled with arrows in the lower portions of Figure 4A (Control) and Figure 4B (Dyslexia), where the decoding-accuracy traces have been reproduced. Arrows in the upper portions of Figures 4A,B label peak latencies. We used bootstrap tests for group differences in latency to compare the corresponding traces across Figures 4A,B.

No group differences were identified at any level of repetition, neither for onset latencies (1sts vs. 2nds, *p* = 0.82; 1sts vs. 3rds, *p* = 0.25; 1sts vs. 4ths, *p* = 0.46), nor for peak latencies (1sts vs. 2nds, *p* = 0.50; 1sts vs. 3rds, *p* = 0.50; 1sts vs. 4ths, *p* = 0.89). This indicates that the multivariate mismatch has a similar time course in both groups, both in terms of when it becomes statistically reliable and when it reaches its peak classification accuracy. Across groups and levels of repetition, information in the neural response that distinguished standards from deviants arose between 96 and 145 ms and peaked between 257 and 315 ms with respect to sound onset.

### Repetition Strengthens the Standard Representation in Control

We next asked whether deviant-vs.-standard decoding becomes significantly stronger as the number of standard repetitions increases from two to three to four. Within each group separately, we performed three one-sided sign-permutation tests (cluster-defining threshold *p* < 0.05, corrected significance level *p* < 0.05) on all post-stimulus time points in the decoding-accuracy time series (1sts vs. 3rds > 1sts vs. 2nds; 1sts vs. 4ths > 1sts vs. 2nds; 1sts vs. 4ths > 1sts vs. 3rds).

The overlaid time series suggest that decoding accuracy increases with the number of standard repetitions in Control (Figure 4A) but not Dyslexia (Figure 4B). Across all six tests, one significant cluster was identified, indicating that 1sts-vs.-4ths decoding was significantly more accurate than 1sts-vs.-2nds decoding in the Control group (*p* = 0.0004; Figure 4A). No clusters were identified in the Dyslexia group (Figure 4B). This pattern of results was robust to reducing the number of available trials by two-thirds, following the procedure that yielded Figure 3B: 1sts-vs.-4ths decoding was, again, significantly more accurate than 1sts-vs.-2nds decoding in the Control group (two clusters: *p* = 0.001 and *p* = 0.04; Figure 4C), and no significant differences were identified in the Dyslexia group (Figure 4D). Since the deviant 1sts are held constant across the three tests in each group, these results suggest that, in Control, additional repetitions yield more reliable information about standardness (such that standard trials are reliably different from deviant trials, reflected in the performance of a cross-validated standard-vs.-deviant classifier), while in Dyslexia, there is no effect of repetition level on the multivariate mismatch.

### Standard-vs.-Deviant Information Arises at Similar Latencies Across Levels of Repetition

Having seen that repetition can strengthen the standard representation (Henson, 2003), we then asked if it can also facilitate its processing in time. We evaluated the hypothesis that standards with more repetitions would have shorter decoding latencies, and that this too might differ between groups. Within each group separately, and for onsets and peaks separately, we used bootstrap tests to compare the latencies at three levels of repetition [(1sts vs. 3rds) vs. (1sts vs. 2nds); (1sts vs. 4ths) vs. (1sts vs. 2nds); (1sts vs. 4ths) vs. (1sts vs. 3rds)].

No significant effects of the standard’s repetition history were found in either Control (Figure 4A) or Dyslexia (Figure 4B), neither for onset latency nor for peak latency (*p*’s between 0.13 and 0.94). Thus, these data provide no evidence that the latency of the multivariate mismatch is affected by the repetition history of the standard.

### Identifying Stimulus-Specific Multivariate Mismatches Depends Critically on Trial Numbers

In a final analysis, we aimed to determine whether the multivariate mismatch shows stimulus specificity. Because the roving-oddball paradigm (Figure 1) sampled each participant’s perception of the /ba/-/da/ continuum, we decoded standards from deviants as a function of their acoustic-phonetic distance, hypothesizing that the closer the two stimuli, the smaller the deviant response and thus the less decodable they would be. Prior studies have shown parametric effects of deviance magnitude on the MMN (Jaramillo et al., 2000; Joanisse et al., 2007; Pakarinen et al., 2013). We also considered the possibilities that phonological category structure, not strictly acoustic-phonetic distance, would influence the mismatch responses, and/or that this would differ in Dyslexia, given prior reports of abnormal speech-sound discrimination in this population (Werker and Tees, 1987; Bogliotti et al., 2008).

We conducted 25 separate decoding analyses, pairing each of five possible standard stimuli (defined as the 4th in its train) with each of the five possible stimuli that could follow it (itself as the 5th in the train, or any of the other four stimuli as subsequent deviants). In this way, we precisely controlled the stimulus history of the standard as well as the phonetic distance between it and the deviant. This came at the cost, however, of severely reducing the number of trials available for training and testing the classifier. Specifically, before artifact rejection, there were 120 trials per standard and no more than 14 trials per deviant. In the most extreme example, due to stimulus pseudo-randomization, there were only 5 occurrences of four E’s (standard) followed by a B (deviant).

**FIGURE 5:**
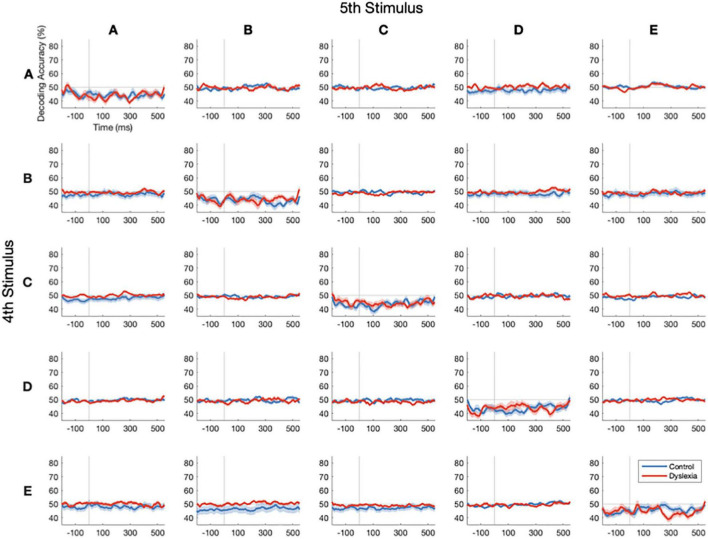
Stimulus-specific standards vs. deviants cannot be decoded with low numbers of trials. Decoding-accuracy time courses for stimulus-specific standard-to-deviant transitions in Control (blue) and Dyslexia (red). Rows indicate the standard stimulus (the 4th in its train) and columns indicate the stimulus that followed it in time, either a deviant 1st or a 5th in the same train. Reliable decoding of standards and deviants above the 50% chance level could not be achieved with the low numbers of stimulus-specific transitions in our paradigm. Along the diagonal, the classification of identical stimuli yields below-chance classification performance.

As depicted in Figure 5, reliable decoding of standards vs. deviants above the 50% chance level could not be achieved with these numbers of trials, neither in Control nor in Dyslexia. This parallels the results in Figure 3, in which subsetting the trials yielded lower decoding accuracies. Along the diagonal, attempts to classify 4th vs. 5th presentations of identical stimuli yield below-chance performance. Simulations indicate that this is likely to occur when cross-validated linear classification is performed on biological data that are low in both sample size and effect size (Jamalabadi et al., 2016), as we would expect for physically identical standards. In sum, we found that these data were not suitable for comparing multivariate mismatch responses as a function of stimulus differences due to the low number of trials for each specific standard-to-deviant transition.

## Discussion

### Summary of Results

The neural representation of a repeated stimulus is the standard against which a deviant stimulus is measured in the brain. The two principal aims of this study were to determine whether neural responses are less sensitive to repetition in dyslexia, and whether repetition builds an increasingly robust standard in dyslexia, as we hypothesized it would in typical readers. We first demonstrated that multivariate decoding of MEG data recorded during an auditory roving-oddball paradigm can differentiate deviant trials from standard trials – a “multivariate mismatch” – despite no stimulus differences between them. We then showed that, in both groups, standards generated by as few as two and as many as six repetitions were distinct from deviants, inconsistent with the hypothesis that the brain is less sensitive to repetition in dyslexia. However, we found evidence that repetition builds a standard that is increasingly different from a deviant in the typical-reader group only. Throughout these analyses, we identified no differences in decoding latency between the groups, inconsistent with the idea that cortical deviance detection is delayed in dyslexia. Together, these results suggest that many of the neural mechanisms that give rise to the change-detection/regularity-violation response are intact in dyslexia, with the possible exception of a putatively predictive mechanism that is sensitive to the quantity of prior sensory information.

### Comparison of Univariate and Multivariate Mismatch Responses

The present study differs from numerous prior investigations of the MMN/MMF in dyslexia [reviewed in Näätänen et al. (2012)] because of its multivariate rather than univariate approach to identifying differences between deviant and standard neural responses (To facilitate comparison with other studies, topographical plots and waveforms of univariate sensor data are provided in Supplementary Figure 1). While univariate analysis follows an activation-based philosophy – looking for monotonic increases in neural activity as a function of experimental variables – multivariate analysis exploits *any* difference between experimental conditions to conclude that the brain contains information about the contrast of interest (Hebart and Baker, 2018). Multivariate decoding can succeed on the basis of spatially distinct neural populations fluctuating in non-uniform directions, and thus it is generally considered a more sensitive technique, usually at the cost of less interpretability in the standard univariate framework. That is all to say that the multivariate mismatch may or may not bear any resemblance to the more familiar MMN and MMF components.

However, the multivariate mismatch, which we found to peak at 282 ms after sound onset, appears not dissimilar in timing and morphology to other speech-evoked MMFs. For example, Kujala et al. (2004) identified a sublexical, phonological mismatch response that originated in left temporal cortex and peaked between 280 and 300 ms. Paul et al. (2006) presented near-boundary /ba/ and /da/ tokens in an oddball paradigm to 9-year-old children with and without dyslexia. The difference between deviant and standard amplitudes was measured at left fronto-temporal sensors within three time windows that they felt best captured the component morphology: the rising slope at 180–230 ms, the peak at 230–280 ms, and the falling slope at 280–330 ms. As can be appreciated from Figure 2A, these are also apt descriptors for the time course of decoding accuracy in our study, and like us, Paul et al. (2006) found no difference in the mismatch between typical readers and individuals with dyslexia. Given the similarities between these paradigms and results, it is possible that the two analytic approaches have identified the same neural phenomena.

A second important methodological note is that the auditory mismatch brain response has generators in the temporal lobes, bilaterally, and the frontal lobes, with a right-hemisphere bias (Giard et al., 1990). The temporal component is associated with pre-attentive change detection, while the frontal component is associated with attentional reorienting and conscious processing. However, because MEG is insensitive to cortical sources that are oriented radially (i.e., toward or away from the scalp, versus tangentially, or parallel to the scalp), it captures the temporal-lobe component originating from within the Sylvian fissure but not the frontal component (Hämäläinen et al., 1993; Näätänen et al., 2012). Therefore, a conservative interpretation of the multivariate mismatch reported in the present study is that it reflects, primarily or exclusively, the standard-vs.-deviant information contained in the activity of bilateral auditory cortex. It is thus notable that discriminating information lasted for more than 400 ms. The temporal generalization analysis further demonstrated that the neural signature of deviance is dynamically changing, consistent with a hierarchical propagation of prediction errors throughout this region (King et al., 2014).

### Dishabituation, Prediction Error, and Implicit Learning

Indeed, the prevailing mechanistic view of the mismatch response is that it represents prediction error (Näätänen et al., 2005; Wacongne et al., 2012; Parras et al., 2017). As such, the conditions that provoke a mismatch are thought to be the result of an active sensory-learning process, mediated by NMDA-dependent plasticity (Javitt et al., 1996; Wacongne et al., 2012), rather than passive synaptic habituation (cf. May and Tiitinen, 2010). However, the multivariate mismatch is agnostic to the mechanism of mismatch generation. The classification results could certainly be obtained on the basis of feedforward habituation of sensor amplitudes, such that neural responses in both groups habituate from the second repetition, but control participants demonstrate a cumulative reduction over time. In this framework, the notion of “the neural representation of a standard” might refer not to a qualitatively distinct representation of an expected sensation, but simply to a habituated, lower-amplitude deviant response.

More likely, however, is that the representation of a standard stimulus arises though a combination of the aforementioned feedforward repetition effects and feedback-mediated predictive processes. The latter may be instantiated in circuits that interface between descending predictions and ascending stimulation. Here, the progressive attenuation of prediction error over repetitions reflects gradual refinements of predictions as these circuits successively integrate recent sensory information (Auksztulewicz and Friston, 2016). Thus, perceptual learning occurs across stimulus repetitions as feedforward processing is guided by plastic, predictive models. In this framework, successful decoding of deviants from standards across levels of repetition may be due to intact feedforward adaptation processes in both groups, while an additional, parametric effect of repetition on accuracy is due to a predictive mechanism that is more efficacious in controls. We favor this interpretation based on the results of a recent study from our group (Beach et al., 2022). The goal of that study was to identify the source of neural adaptation deficits (e.g., Perrachione et al., 2016) in dyslexia. We orthogonally manipulated the expectation of stimulus repetition and stimulus repetition itself, then measured event-related potentials to unexpected repetitions (reflecting feedforward repetition suppression), expected repetitions (reflecting stimulus-specific predictions), and unexpected changes (reflecting prediction error). We found that while feedforward repetition effects were similar in controls and dyslexia, prediction error was significantly reduced in dyslexia. Consistent with the current study, this suggests that manifestations of rapid plasticity that rely on consistent reactivation of the same neural populations *via* stimulus repetition are intact in dyslexia [Likewise, in the original study (Perrachione et al., 2016), low levels of neural adaptation were still measured in individuals with dyslexia]. However, an available prediction appears to have less of an effect on feedforward processing in dyslexia than it does in typical readers. The larger consequences of a deficit in expectation integration (Beach et al., 2022) may include reduced perceptual efficiency and diminished learning signals (i.e., prediction error) during perceptual processing.

These interpretations are in line with a growing literature on implicit learning deficits in dyslexia. These reports span domains, including motor learning (Lum et al., 2013), visual statistical learning (Sigurdardottir et al., 2017), visual noise exclusion (Sperling et al., 2005), auditory category learning (Gabay and Holt, 2015), and auditory perceptual learning (Ahissar et al., 2006). A common thread among these lines of investigation is that stimulus regularities provide an opportunity to generate predictions. Ideally, expected input should be processed more efficiently, while mismatches should trigger an error response that, in a virtuous circle, improves future predictions (Press et al., 2020). In dyslexia, however, the availability of predictions seems to have a reduced effect on perception, and this may be related to findings, in other studies, of reduced neural mismatch responses in dyslexia (Hämäläinen et al., 2013; Gu and Bi, 2020). On the other hand, the overall quality of the MMN evidence for reduced automatic discrimination of speech in dyslexia has been criticized (Bishop, 2007). Regarding conflicting results on the mismatch in dyslexia, it may prove useful to dissociate speech-perception variables from regularity-detection variables, and, further, to relate neural indices to behavioral discrimination measures and wider difficulties with oral and/or written language (e.g., Tuomainen, 2015).

### Caveats

A clear limitation of this study is that there were insufficient numbers of trials to investigate the stimulus specificity of the multivariate mismatch, and whether this varied between control and dyslexia. Given the field’s longstanding interest in possible speech-perception deficits in dyslexia, it would have been desirable to quantify the neural dissimilarity of between- and within-category tokens from the /ba/-/da/ continuum as revealed by stimulus-specific decoding accuracy, and to directly compare those results with ones from the extant MMN/MMF literature on dyslexia. Future work may succeed in designing a more sophisticated stimulation paradigm – or simply a longer one – with adequate power to detect any stimulus-specific effects. One challenge in this area is that standard effect-size measures are not easily derived from multivariate decoding accuracies (Hebart and Baker, 2018).

A related point is that individual differences in speech perception may have muddied the reported effects. For example, an individual with underspecified phonemic representations might show a smaller mismatch response to standard and deviant stimuli from different linguistic categories (e.g., stimuli A and E). An individual with atypically good discrimination of within-category speech tokens (e.g., stimuli A and B, or stimuli D and E) might show a larger mismatch response to these pairings. An individual with an underlying auditory sensory impairment, particularly one related to rapid spectro-temporal processing, might show less of a mismatch to *any* change in stimulation along the /ba/-/da/ continuum, as these tokens are distinguished by brief (∼40-ms) acoustic transitions between the consonant and the vowel. All of these “individual” profiles have been advanced as characteristics of at least a subset of people with dyslexia (Tallal, 1980; Godfrey et al., 1981; Serniclaes et al., 2004). Therefore, each of the main findings in this paper is tempered with the knowledge that some deviants may or may not have been perceived or encoded as such. But on the other hand, this makes the series of positive results in the dyslexia group even more convincing – that is, we identified robust and timely mismatch responses in this group, despite putative heterogeneity in auditory perceptual abilities.

Finally, we also investigated a general hypothesis about the efficiency of predictive perception, but suspect that our experimental and statistical approaches were not optimized for this analysis. Prior work in the visual domain found that stimulus-specific expectations induced decodable representations even before stimulus onset (Kok et al., 2017). This result inspired us to ask whether the onset latency and/or peak latency of significant standard-vs.-deviant decoding shortens with increasing repetition, presumably as increasingly reliable predictions pre-activate the expected neural code of the standard. However, we did not find evidence that the latency of the multivariate mismatch is affected by the repetition history of the standard. The fact that speech is a complex stimulus that unfolds over time (as opposed to a static visual grating that can be perceived instantaneously) may reduce the inherent temporal precision of the emergence of speech representations in MEG data. Future studies could perhaps create distinct cue and stimulus periods during stimulation for better control.

### Conclusion

We used multivariate MEG decoding to identify the neural signature of standard vs. deviant speech syllables in adults with and without dyslexia. We found no deficit in dyslexia in the immediate sensitivity to stimulus repetition, nor any difference in the latency of standard-vs.-deviant information, both of which likely rely on a combination of feedforward and feedback-mediated perceptual mechanisms. However, we also found evidence that increasing repetition history makes a stronger standard in typical readers, but not in dyslexia. These results are consistent with the idea that, in dyslexia, there is a specific deficit in accumulating short-term statistical regularities and integrating them into perception to improve performance and reduce neural processing costs.

## Materials and Methods

### Participants

Individuals with dyslexia (*n* = 24; 14 female, 10 male; age 18–38 years, mean ± standard deviation = 27 ± 6) and typical readers (*n* = 24; 11 female, 13 male; age 19–41 years, mean ± standard deviation = 26 ± 6) participated in this study. All were right-handed, native speakers of American English with a standard score above 85 on the Matrices subtest of the Kaufman Brief Intelligence Test – Second Edition (KBIT-2; Kaufman and Kaufman, 2004). To confirm self-reports of normal hearing, we conducted pure-tone audiometry tests at the standard frequencies of 250, 500, 1,000, 2,000, 4,000, and 8,000 Hz; all thresholds were ≤35 Hz. For inclusion in the Dyslexia group, participants were required to score below 90 (where the mean standard score is 100 and the standard deviation is 15) on at least two out of four of the following single-word reading measures: Word Identification (untimed real words) and Word Attack (untimed pseudowords) subtests of the Woodcock Reading Mastery Tests – Third Edition (WRMT-III; Woodcock, 2011) and Sight Word Efficiency (timed real words) and Phonemic Decoding Efficiency (timed pseudowords) subtests of the Test of Word Reading Efficiency – Second Edition (TOWRE-2; Torgesen et al., 2012). Control-group participants scored 90 or above on all four of the measures. Table 1 provides a behavioral characterization of the two groups. By design, the Dyslexia group scored significantly below the Control group on single-word reading. They also scored significantly lower on verbal working memory, oral reading of connected text, and two out of three measures of phonological processing. Eighteen individuals in the Dyslexia group reported a prior formal diagnosis of dyslexia. All participants gave written, informed consent as overseen by the Committee on the Use of Humans as Experimental Subjects at the Massachusetts Institute of Technology.

### Stimuli

Stimuli were drawn from a /ba/-/da/ acoustic continuum constructed by Stephens and Holt (2011) from natural-speech endpoints produced by an adult male speaker of English. We selected, for each participant, a set of five stimuli (A–E) that best represented their categorical perception of the continuum (Figure 1A). Prior to the roving-oddball experiment, participants had performed a separate MEG task in which they labeled 40 tokens each of the ten odd-numbered steps of the 20-step Stephens and Holt (2011) continuum, renumbered for our purposes as 1 (/ba/) through 10 (/da/). The tokens were pseudo-randomized and the task was self-paced with no feedback (see Beach et al., 2021 for a full description). During the break between tasks, we fit a logistic function to each participant’s *ba*/*da* response ratio. The continuum step nearest the function’s inflection point was chosen as stimulus C. A and B were selected from the/ba/end of the continuum, and D and E were selected from the/da/end of the continuum. A–E were made equidistant from one another. In practice, there was little variation across individuals in the categorical perception of the continuum: for 21 of the 48 participants, step 5 was the most ambiguous, and for the other 27 participants, step 6 was the most ambiguous. Therefore, 21 participants (12 Control and 9 Dyslexia) subsequently heard the odd-numbered stimuli and 27 participants (12 Control and 15 Dyslexia) heard the even-numbered stimuli in the roving-oddball paradigm.

Stimuli A–E were then presented in a roving-oddball design made up of 600 consecutive “trains” of four, five, or six identical stimuli (Figure 1B). The length and order of trains within the design was pseudo-randomized such that all possible stimulus transitions occurred (e.g., five B’s followed by four C’s, four E’s followed by six A’s, etc.) and were roughly equiprobable. The task design was the same for all participants. Syllable duration was 310 ms and stimulus onset asynchrony was 575 ms, both within and between trains. In all, 3,000 stimuli were presented: 600 that were the 1st token in their train (i.e., deviants, representing a change in stimulus from the previous train), 600 “2nds,” 600 “3rds,” 600 “4ths,” 400 “5ths,” and 200 “6ths.”

### Procedure

Behavioral assessment (Table 1) was performed by an experienced tester on a separate day prior to MEG recording. Assessments were audio-recorded and scored for reliability by a second tester.

During the MEG session, participants were passively exposed to the stimuli while they watched a silent movie (*Wall-E*) for approximately 28 min. Movie onset was jittered across participants to prevent time-locked visual or semantic information from contaminating the MEG signal. Auditory stimuli were delivered over insert earphones (Etymotic Research, Inc., Oak Grove, IL, United States) at a comfortable listening level fixed across participants. Participants were told that they would hear sounds that could be ignored and were asked to keep their eyes open during the experiment. No responses were required.

### Magnetoencephalography Recording and Preprocessing

Magnetoencephalography was recorded using an Elekta Triux 306-channel system comprising 102 magnetometers and 204 planar gradiometers, with a sampling rate of 1,000 Hz and online filtering between 0.03 and 330 Hz. Continuous measurements of head position were made with five coils attached to the scalp. Prior to recording, anatomical landmarks (nasion, left preauricular, and right preauricular) were registered with respect to the head-position coils using a Polhemus digitizer (Colchester, VT, United States). Maxfilter Software (Elekta, Stockholm, Sweden) was used to correct for head movement and filter out noise sources originating from outside the MEG helmet. Using Brainstorm software (Tadel et al., 2011), eye-blink and cardiac artifacts were removed from each participant’s continuous dataset *via* signal-space projection. Trials were epoched from −200 to 550 ms with respect to stimulus onset. Trials with zero signal or in which any sensor exceeded a peak-to-peak amplitude of 10,000 fT (for magnetometers) or 2,500 fT/cm (for gradiometers) were excluded from further analysis. Trials were then low-pass filtered at 15 Hz. Finally, sensor amplitudes were z-normalized for the subsequent multivariate pattern analysis using the mean and standard deviation of the prestimulus period (−200 to 0 ms).

### Multivariate Pattern Analysis

Pattern classification was performed using linear support vector machines (SVM) as implemented in LIBSVM 3.21 (Chang and Lin, 2011) for MATLAB (MathWorks, Natick, MA, United States). SVM classification was performed for each participant separately and at each time point independently, and, in all cases, to distinguish data from two conditions (e.g., 1sts vs. all standards, 1sts vs. 4ths, etc.). The data consisted of vectors of the 306 sensor measurements at each time point, extracted from each trial of the two conditions under study.

We used a cross-validation procedure in which the data were randomly assigned to one of five folds; four folds were used for training the classifier and one fold was used for testing it. To equalize the noise level across the data, we used the “epoch” method of multivariate noise normalization (Guggenmos et al., 2018), whereby the noise covariance matrix is computed for all time points in the epoch separately within each condition and then averaged across time points and conditions. To guard against inflated classification, the noise covariance was estimated from the training folds and applied to both the training folds and the test fold. Additionally, because the estimate of noise covariance can be unstable when there is relatively little data with respect to the number of features (i.e., sensors), we applied the shrinkage transformation (Ledoit and Wolf, 2004) to regularize the estimate and prevent overfitting. To improve the signal-to-noise ratio, trials from the same condition within each of the five folds were averaged, yielding one summary trial per condition per fold. The entire decoding procedure was repeated 100 times, yielding one averaged decoding-accuracy time series per participant in which 50% accuracy is considered chance performance of the classifier.

### Statistical Inference

Significance of decoding-accuracy time courses was determined by non-parametric sign-permutation tests applied to the time window of 0–550 ms. Due to the rapid stimulus presentation, baseline (prestimulus) time was excluded from analysis because it was otherwise tested at the end of other trial epochs: e.g., 2nds vs. 3rds at −100 ms is equivalent to 1sts vs. 2nds at 475 ms. Permutation samples were created by randomly flipping the participant-specific time courses around the null value (50% for accuracies and 0 for differences in accuracy) and then averaging across participants. Five thousand repetitions of this procedure produced an estimate of the empirical distribution of decoding accuracy with which the true time courses were converted into *p*-value maps. The familywise error across time points was controlled using cluster-based inference: using a cluster-defining threshold of *p* = 0.05, suprathreshold clusters (i.e., contiguous time points) were first identified and then reported as significant if the sum of their within-cluster values exceeded a *p* = 0.05 threshold with respect to the empirical distribution of the suprathreshold clusters of the permuted statistical maps.

### Temporal Generalization Analysis

To determine the stability of representational neural codes over time, we employed the temporal generalization approach to multivariate pattern analysis (King and Dehaene, 2014). To reduce computational load, MEG data were downsampled by a factor of 4 for this analysis. As before, SVM classifiers were trained at each time point, but then tested at every time point in the dataset. If the neural patterns that distinguish the two classes (here, deviants and all standards) are stable over time, then the classifier should successfully generalize to other time points. Results are depicted in a matrix, averaged over participants, with training time on the *x*-axis and testing time on the *y*-axis. Regions of significant temporal generalization between 0 and 550 ms were determined by sign-permutation tests as described above.

### Onset and Peak Latency Analysis

To determine whether the onset and/or peak latencies differed between groups (Control vs. Dyslexia) and/or by repetition (e.g., 1sts vs. 3rds vs. 1sts. vs. 2nds), we conducted bootstrap tests (Cichy et al., 2017; Mohsenzadeh et al., 2018). We bootstrapped the participant-specific decoding-accuracy time series 1000 times to obtain (a) onset latencies of significant decoding (determined by sign-permutation tests as described above) and (b) latencies of peak decoding accuracy, each restricted to the period between 0 and 550 ms, such that we could then calculate an empirical distribution of latency differences for each between-group or within-group comparison of interest. We calculated a two-sided *p*-value by dividing the number of latency differences that were smaller (or larger, as appropriate) than zero by the number of bootstrap samples.

## Data Availability Statement

The raw data supporting the conclusions of this article will be made available by the authors, without undue reservation.

## Ethics Statement

The studies involving human participants were reviewed and approved by the Committee on the Use of Humans as Experimental Subjects at the Massachusetts Institute of Technology. The participants provided their written informed consent to participate in this study.

## Author Contributions

SB, OO-P, TC, TP, and JG conceived and designed the study. SB, OO-P, and SM collected the data. DP developed analysis code. SB analyzed the data and wrote the manuscript. All authors approved the submitted version.

## Author Disclaimer

The content is solely the responsibility of the authors and does not necessarily represent the official views of the NIH.

## Conflict of Interest

The authors declare that the research was conducted in the absence of any commercial or financial relationships that could be construed as a potential conflict of interest.

## Publisher’s Note

All claims expressed in this article are solely those of the authors and do not necessarily represent those of their affiliated organizations, or those of the publisher, the editors and the reviewers. Any product that may be evaluated in this article, or claim that may be made by its manufacturer, is not guaranteed or endorsed by the publisher.
