# Supplementary material for: The Neural Representation of a Repeated Standard Stimulus in Dyslexia
Source: Front Hum Neurosci. 2022 May 12;16:823627. doi: 10.3389/fnhum.2022.823627 (PMC9133793; doi:10.3389/fnhum.2022.823627)
Supplement: Supplementary file 1 [file Data_Sheet_1.PDF]

Supplementary Material

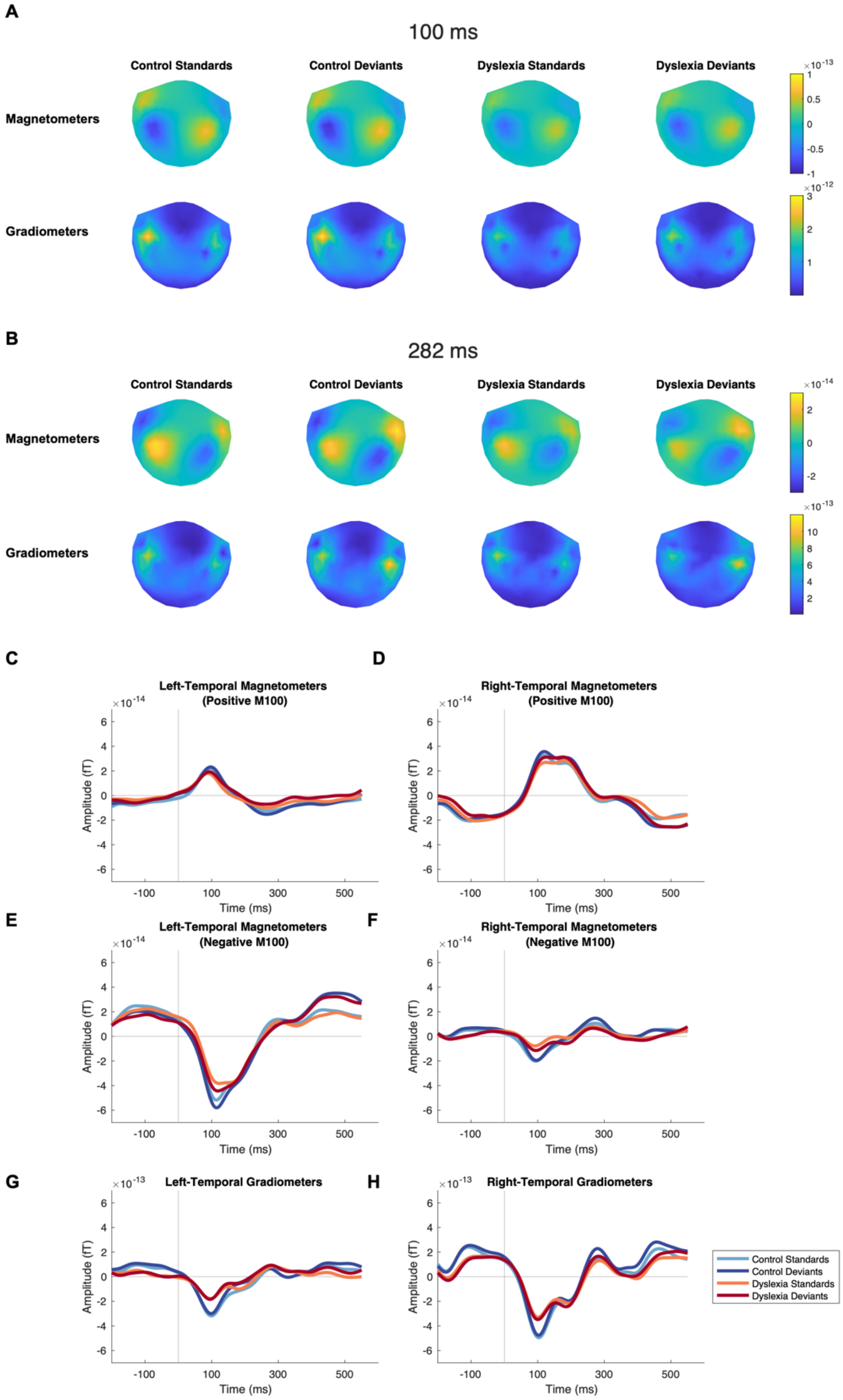

Supplementary Figure 1. Univariate sensor amplitudes for standards and deviants in Control and Dyslexia. Topographical plots of sensor amplitudes at **(A)** 100 ms (M100 evoked response) and **(B)** 282 ms (peak standard-vs.-deviant decoding across all participants, as in **Figure 2A**). Left is left and top is anterior. Magnetometers (top row) and gradiometers (bottom row) are plotted separately. The scale (fT) is constant across each row, showing that the univariate neural activity for deviants (1sts) and standards (2nds through 6ths), and for Control and Dyslexia, differs somewhat in degree but little in location. Likewise, waveforms have similar morphology in Control and Dyslexia: **(C)** left-temporal magnetometers with a positive M100 (n = 8); **(D)** right-temporal magnetometers with a positive M100 (n = 7); **(E)** left-temporal magnetometers with a negative M100 (n = 5); **(F)** right-temporal magnetometers with a negative M100 (n = 6); **(G)** left-temporal gradiometers (n = 26); **(H)** right-temporal gradiometers (n = 26).
